# Brain Dead or Alive: A Case Report of Inaccurate Neurological Prognostication

**DOI:** 10.7759/cureus.75552

**Published:** 2024-12-11

**Authors:** Danielle A Bazer, Matthew Orwitz, Nicholas Koroneos, Ryan Corn, Phillip Yeung

**Affiliations:** 1 Neurology/Neuro-Oncology, Johns Hopkins University, Baltimore, USA; 2 Neurology, Stony Brook University, Stony Brook, USA

**Keywords:** ancillary testing, brain death, case report, neuro-prognostication, nonconvulsive subclinical seizures

## Abstract

Although numerous definitions of brain death exist, the diagnosis and diagnostic process remain open to interpretation. We present the case of a 32-year-old male with systemic lupus erythematosus who presented to an outside hospital following a cardiac arrest while jogging. His electroencephalogram (EEG) showed abnormal contour in the posterior fields. Despite the patient's normal brain magnetic resonance imaging (MRI), the treating neurological team diagnosed him with anoxic brain injury based on his persistently comatose exam and abnormal EEG. With neurological guidance, the patient’s family elected for terminal extubation and organ donation. He surprisingly survived the terminal extubation and was transferred to our hospital for prognostication. His EEG showed lateralized periodic discharges, prompting medication adjustments. He was also treated for multiple infections. With treatments, his EEG normalized, and he ultimately ambulated, conversed, and consented for this case report one year following discharge. The lack of uniformity on how to approach comatose patients with presumed irreversible neurologic injury can lead to inaccurate prognostication and guide life-or-death clinical decisions. This case of erroneous assessment highlights the marked limitations of the current legal framework for determining brain death and the need for standardized medical criteria.

## Introduction

In 1981, the Uniform Determination of Death Act (UDDA) was approved by the American Medical Association. Part 1 of the law discusses deaths secondary to cardiopulmonary failure, which accounts for the majority of deaths. Part 2 is centered around “death based upon irreversible loss of all brain function,” specifying brain stem and neocortex [1]. The UDDA does not discuss the studies required to ascertain such a diagnosis. The American Academy of Neurology (AAN) utilizes this law as a medical and legal framework [2].

In 1995, the AAN constructed guidelines defining brain death as “the irreversible loss of function of the brain, including the brain stem” [3]. Although the 1995 AAN guidelines were an attempt to standardize the practice of diagnosing brain death, they did not mitigate the variation of individual institutional protocols and procedures [4,5].

The key to making the brain death diagnosis is a clinical exam, where formal testing serves as supportive and ancillary [2,6]. Yet, roughly 25% of academic physicians do not receive formal training in performing a brain death examination, leading to immense variability in the approach and determination of brain death [7].

The implication of declaring a patient as brain dead is quite grave, as the patient is both deemed clinically and legally dead [6]. Therefore, it is imperative to rule out reversible causes of a coma, such as infections and status epilepticus, and the physician should be confident in his or her ability to appropriately perform a brain death exam.

This article was previously presented as a meeting abstract at the American Academy of Neurology Annual Meeting in April 2022 [8].

## Case presentation

A 32-year-old, left-handed male with a past medical history of systemic lupus erythematous with gastrointestinal involvement was transferred to our hospital for neurologic prognostication. The patient had originally presented to an outside hospital after an out-of-hospital cardiac arrest while jogging. He was intubated upon arrival at the hospital. He was found to have an anterolateral wall myocardial infarction, for which cardiac catheterization was deferred due to an anticipated poor neurological recovery. He had an unremarkable brain MRI six days post-arrest. Electroencephalogram (EEG) showed sharply contoured waves in the occipital fields. He was started on levetiracetam for antiseizure prevention. His treating team, comprised of multiple specialties including neurologists, assessed him to have a poor neurological status, yet he was never formally declared brain dead, as his cranial nerves were intact. Given the patient’s anticipated poor prognosis, the family decided on a terminal extubation and organ donation fifteen days post-arrest. However, the patient survived the extubation and was soon able to communicate.

**Figure 1:**
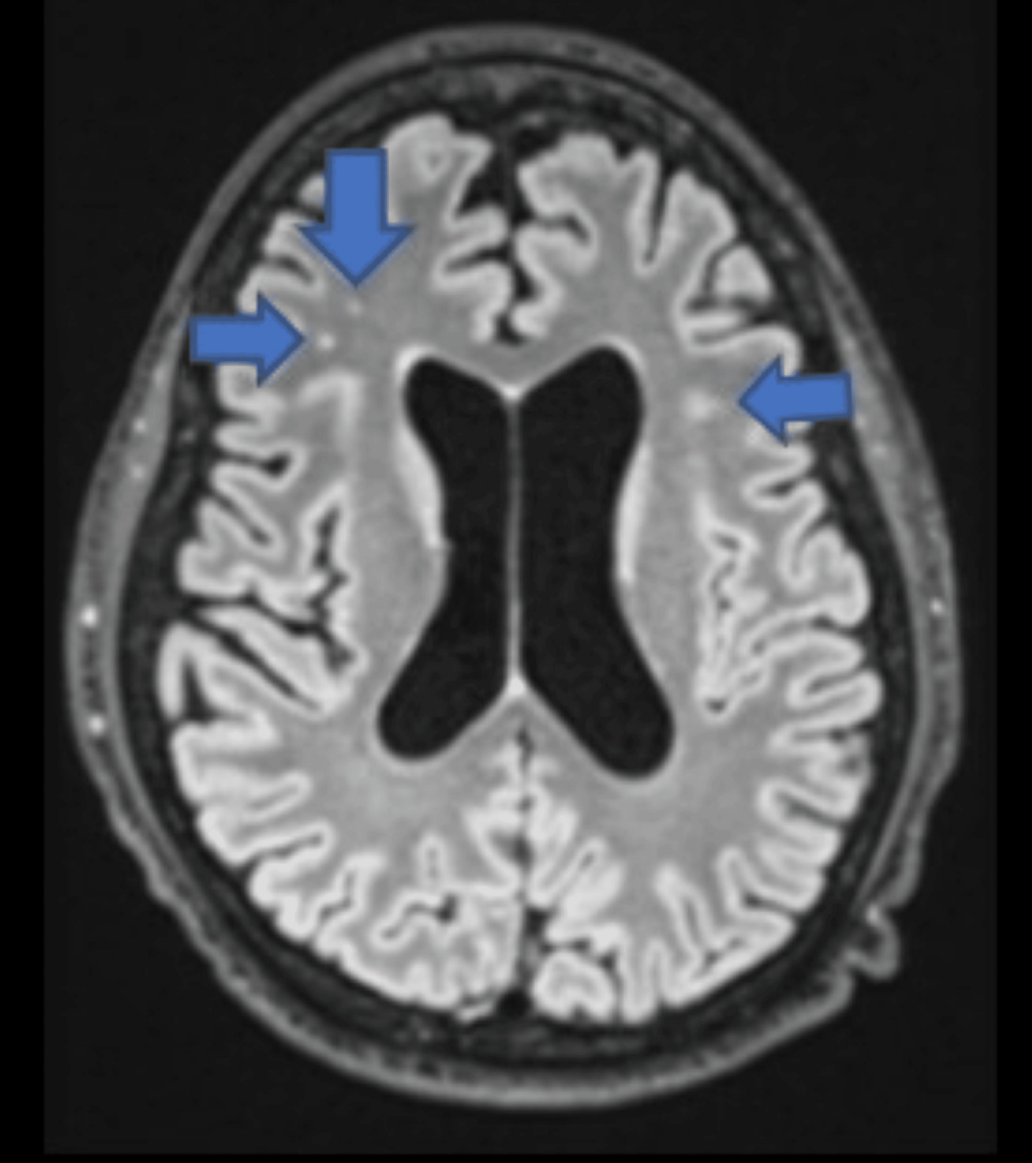
MRI of the brain three weeks post-arrest MRI T2/FLAIR of the brain revealing punctate foci of T2 hyperintensity within the subcortical white matter.

The patient was transferred to our institution at the family's request for neurological prognostication 20 days post-arrest. Upon arrival, he was awake, cranial nerves were intact, and he was tracking noise and movement. The patient was found to have a urinary tract infection and Clostridium difficile infection. On post-arrest day 22, a subsequent EEG revealed lateralizing periodic discharges, which aligned with clinical myoclonus, prompting an increase in his levetiracetam dosage. He required multiple antiseizure medications, ultimately including clobazam. With the initiation of clobazam, the lateralizing periodic discharges resolved. He became more alert and interactive, and his speech became coherent. He later moved his extremities to command. MRI of the brain with gadolinium revealed punctate foci of T2 hyperintensity within the subcortical white matter, consistent with anoxic brain injury (Figure 1).

**Figure 2:**
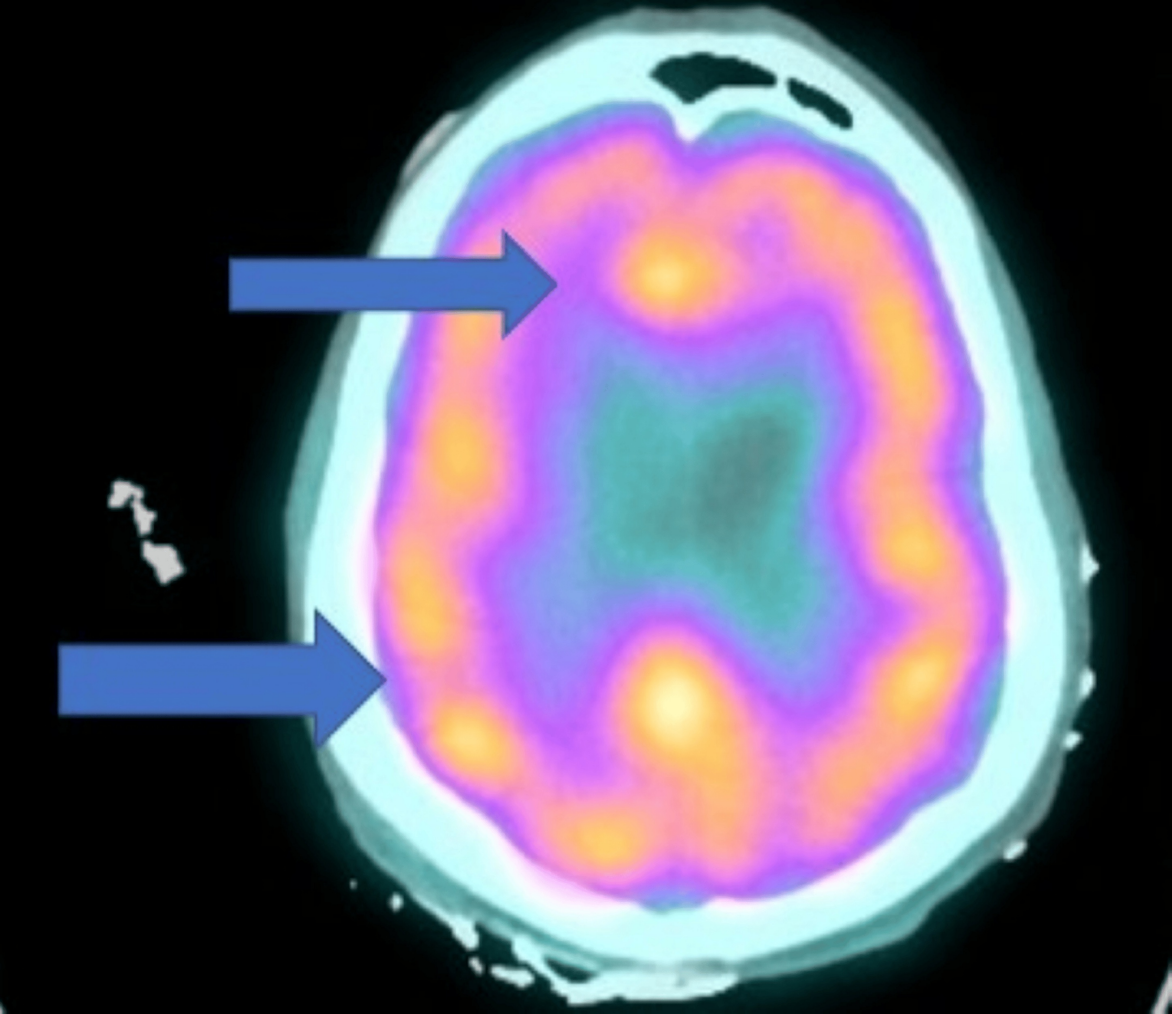
Brain single-photon emission computed tomography Brain single-photon emission computed tomography with markedly diminished activity in the anterior corpus callosum and right poster parietal-temporal lobes.

Brain single-photon emission computed tomography (SPECT) scan showed markedly diminished activity in the anterior corpus callosum and right posterior parietal-temporal lobes (Figure 2).

The patient was discharged to traumatic brain injury rehabilitation. He was followed up in our clinic three months post-arrest, at which point he was able to ambulate with a walker and able to participate in the physical exam fully. The patient personally consented to the publication of this case report.

## Discussion

Our patient's prognostication was inaccurate due to a reversible etiology of coma, as he was having intermittent seizures, which were not captured on EEG at the outside hospital. Additionally, his concomitant infections, which were untreated, may have further lowered his seizure threshold, contributing to his intermittent seizures and fluctuating mental status. After treating both the seizures and the underlying infections, the patient had an improved mental status and physical exam. This suggests that if the infections had been treated more promptly, then his neurological status would have been improved, and his anticipated prognosis by the outside hospital would have been vastly different.

It is also important to note that the patient’s original EEG at the outside hospital only showed nonspecific findings of sharply contoured waves in the posterior occipital fields rather than a flat or burst suppression pattern. Taken together with the normal brain MRI six days post-arrest, this suggests that the neurological prognosis may not have been as grim as initially communicated with the patient’s family.

The current literature suggests that firm discussions surrounding neurological prognostics should not be initiated within 72 hours of the traumatic event [9]. Although a comatose state subsequent to the event typically portends a poor outcome and yields guarded neurological prognostication, a comatose state should not dissuade the examiner from performing a thorough work-up [10]. Conversely, an inappropriate neurological prognostication may result in the continuation of life-sustaining treatments, which may ultimately prove futile [11].

It is important to have frequent conversations with a patient’s family regarding the patient’s clinical condition. The concept of brain death is challenging for families; therefore, early conversations regarding the severity of the patient’s condition are imperative to aid in the acceptance of the challenging diagnosis [12]. A multidisciplinary team is best to help guide the patient’s family during the process of declaring brain death [13]. Conversations with the family can and should begin prior to the official declaration of brain death [12].

Our patient’s trajectory delineates the consequences of inaccurate neurological prognostication. The lack of uniformity on how to approach comatose patients with presumed irreversible neurologic injury significantly contributed to the decision-making. Given the limitations of the current legal framework for determining brain death under the UDDA, we are reiterating the importance of ancillary testing and the potential for true life-or-death consequences of inaccurate neurological prognostication.

## Conclusions

Inaccurate neurological prognostication secondary to reversible causes of coma may carry true life-or-death consequences. A thorough work-up is required to ensure that patients are properly diagnosed and treated prior to delivering a neurological prognosis. As a result, when there is ambiguity in a case, particularly in a young individual, ancillary testing is imperative. Ancillary testing should not replace but rather supplement a thorough bedside physical examination.
